# Online near-infrared analysis coupled with MWPLS and SiPLS models for the multi-ingredient and multi-phase extraction of licorice (*Gancao*)

**DOI:** 10.1186/s13020-015-0069-2

**Published:** 2015-12-18

**Authors:** Yang Li, Mingye Guo, Xinyuan Shi, Zhisheng Wu, Jianyu Li, Qun Ma, Yanjiang Qiao

**Affiliations:** School of Chinese Materia Medica, Beijing University of Chinese Medicine, Beijing, China; Pharmaceutical Engineering and New Drug Development of Traditional Chinese Medicine, Ministry of Education, Beijing, China; Key Laboratory of Traditional Chinese Medicine-Information Engineering, State Administration of Traditional Chinese Medicine, Beijing, China; Beijing Key Laboratory for Basic and Development Research on Chinese Medicine, Beijing, China

## Abstract

**Background:**

This study aims to analyze the active pharmaceutical ingredients (APIs) of licorice (*Radix Glycyrrhizae*; *gancao*), including glycyrrhizic acid, liquiritin, isoliquiritin and total flavonoids, in multi-ingredient and multi-phase extraction by online near-infrared technology with fiber optic probes and chemometric analysis.

**Methods:**

High-performance liquid chromatography and ultraviolet spectrophotometry determined the APIs content in different extraction phases by online near-infrared analysis, which included sample set selection by the Kennard–Stone algorithm, optimization of spectral pretreatment methods (i.e., orthogonal signal correction and wavelet denoising spectral correction), and model calibration by the partial least-squares algorithm, moving-window partial least-squares algorithm and synergy interval partial least-squares (SiPLS) algorithm. The relative errors and F values were used to assess the models in different extraction phases.

**Results:**

The root-mean-square error of correction, root-mean-square error of cross-validation and root-mean-square error of prediction of APIs in the SiPLS model was less than 0.07. The F values of glycyrrhizic acid, liquiritin, isoliquiritin and total flavonoids were 10,765, 32,431, 649 and 6080, respectively, which were larger than 6.90 (*P* < 0.01).

**Conclusion:**

The study demonstrated the feasibility of online NIR analysis in the multi-ingredient and multi-phase extraction of APIs from licorice.

**Electronic supplementary material:**

The online version of this article (doi:10.1186/s13020-015-0069-2) contains supplementary material, which is available to authorized users.

## Background

The *Process Analysis Technology Industry Guide* was published by the U.S. Food and Drug Administration for encouraging drug development with the use of online analysis [1]. Process analysis technology is applicable monitoring of raw materials and key intermediates in real time and for quality assurance of the final products.

Near-infrared (NIR) analysis can be applied online as an effective process analysis [1]. Online NIR analysis is coupled with an optical fiber in manufacturing for the online monitoring of critical process parameters that control the quality of production [2].

NIR analysis can be used to identify active pharmaceutical ingredients (APIs) [2, 3]. The technology has also been applied to Chinese medicine (CM) in the extraction of an individual ingredients; e.g., *Ligusticum chuanxiong* (*Chuanxiong*) [4], *Salvia miltiorrhiza* (*Danshen*) [5], *Paeonia lactiflora* (*Shaoyao*) [6] and *Pueraria lobata Ohwi* (*Gegen*) [7]. However, only a few reports mentioned the application of online NIR analysis for multiple ingredients and APIs of low concentration, *e.g.*, *Astragali Radix* (*Huangqi*) [8] and *Radix Paeoniae Rubra* (*Chishao*) [9].

There is a gap to fill in CM process analysis with an online and reliable detection method that can simultaneously detect multiple ingredients in real time. The majority of APIs is usually extracted with water or other solvents for CM. Multiple phases should be applied to accurately observe the extraction process by NIR technology. However, there was no previous work on online NIR analysis demonstrating the simultaneous detection capability for multi-phase extraction in CM.

Licorice (*Radix Glycyrrhizae*) (*Gancao*) is widely used in CM [10]. APIs are taken from extraction of the dried roots and rhizomes of *Glycyrrhiza glabra* (*Gancao*) [11]. The APIs of licorice include flavonoids, saponins, glycyrrhizic acid and liquiritin, according to *Chinese Pharmacopoeia* (2010 Edition). There was no report on the online monitoring of the multi-phase extraction and the multiple ingredients of licorice.

Online NIR technology was applied to collect spectra in a pilot-scale extraction process. Results obtained using the partial least-squares (PLS) algorithm, moving-window partial least-squares (MWPLS) algorithm and synergy interval partial least-squares (SiPLS) algorithm were compared to high-performance liquid chromatography (HPLC) and ultraviolet (UV) spectrophotometry. Common chemometric indicators [i.e., the lowest root-mean-square error of correction (RMSEC), root-mean-square error of cross-validation (RMSECV) and root-mean-square error of prediction (RMSEP)] were used to assess the models and demonstrate reliable analysis [12]. Furthermore, the relative errors and F-values were used in analysis of the extraction of different phases to evaluate the reliability and detection ability of online NIR analysis [13].

This study aims to analyze the APIs of licorice, including glycyrrhizic acid, liquiritin, isoliquiritin and total flavonoids, in multi-ingredient and multi-phase extraction by online NIR technology with fiber optic probes and chemometric analysis.

## Methods

### Materials

Licorice was collected from Guazhou (Gansu, China), and was empirically identified as *Glycyrrhiza uralensis* Fisch. by Dr. Liu Chunsheng (School of Chinese Materia Medica, Beijing University of Chinese Medicine, China). Glycyrrhizic acid of reference standard (No. 111610-201106) and liquiritin reference of standard (No. 110731-201116) were supplied by the National Institutes for Food and Drug Control (Beijing, China), and isoliquiritin of reference standard was supplied by Jiangxi Herbfine Hi-tech Co., Ltd (Jiangxi, China). Acetonitrile (Fisher Scientific, USA) was of HPLC grade and phosphoric acid was of analytical grade (Beijing Chemical Works, Beijing, China). Deionized water was purified by a Milli-Q water system (Millipore Corp., Bedford, MA, USA).

### Processing and sampling of different extraction phases

A 9-kg quantity of licorice was extracted with eight-fold deionized water in a multi-functional extractor (100 L) three times at 2.5-h intervals. The stirring paddle (HCHT System, Beijing, China) was set at a speed of 50 rpm. During the extraction, NIR spectra were scanned periodically (Table S1 in Additional file 1). According to the contents of the four ingredients, a reasonable sampling interval was determined. In the initial heating and boiling phase, the contents of ingredients varied rapidly, and a short sampling interval was set. As the contents of ingredients varied less in the second and third extractions than in the first extraction, the sampling interval was lengthened to reduce the amount of work in the second and third extractions.

**Fig. 1:**
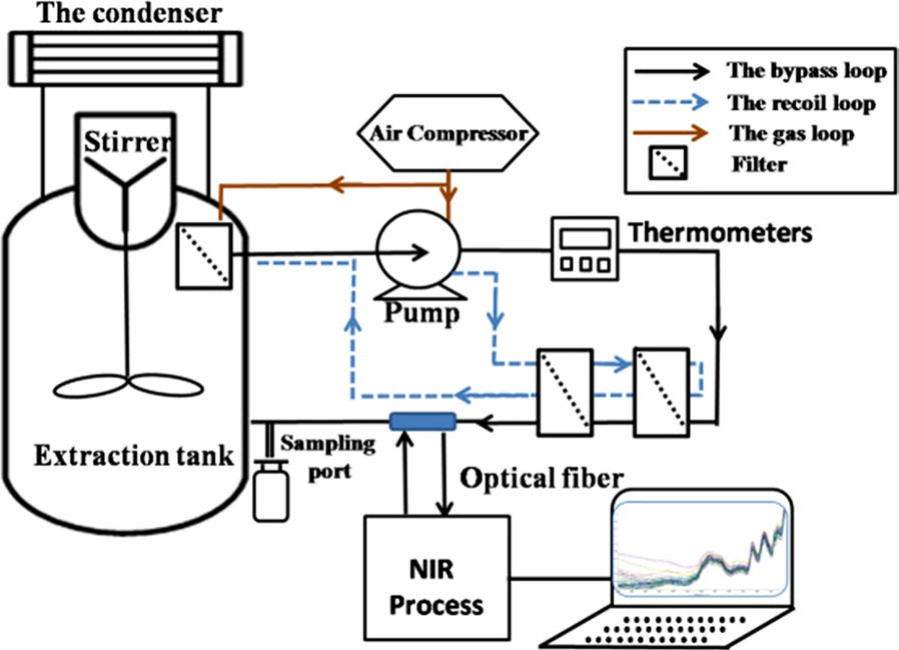
Platform of extraction

The system included an online NIR scanning instrument (Fig. 1). Licorice was added to the tank and extracted with deionized water. Bubbles were eliminated in the bypass pipe by completely submerging the filter in the tank, which was interlinked with the bypass pipe. The extraction solution was circulated in the bypass under the action of a pump. The pump was powered by compressed air provided by an air compressor to eliminate contamination. The 80- and 100-μm filters were used to eliminate the interference from solid content when the extraction solution passed through the bypass [14, 15]. The pump was turned on for 30 s to update the solution in the bypass. The sample was scanned in a flow cell by an optical fiber to ensure samples were in the same environment as the solution in the tank [14]. The recoil loop that reduced the risk of the bypass clogging and eliminated bubbles in the pipe was included.

The temperature was recorded in real time by thermometers (HCHT System, Beijing, China). Throughout the extraction process, spectra were recorded by an online NIR instrument with an optical fiber. As soon as the scanning was completed, the sampling tap was opened and 10 mL of extract solution was collected for HPLC and UV analysis.

### NIR equipment and measurement

Online NIR spectra were collected by fiber optic probes. NIR radiation was applied through a 2-mm optical path using an XDS process analyzer and VISION software (Foss NIR System, Silver Spring, MD, USA). The wavelength range of spectra was between 800 and 2200 nm. Spectra were obtained from an average of 32 scans with a wavelength increment of 0.5 nm.

### HPLC methods

All samples were diluted with 70 % (v/v) ethanol–water solution and the contents of glycyrrhizic acid, liquiritin and isoliquiritin were determined by a reversed-phase HPLC assay with analytical validation. Chromatographic analysis was performed by a Waters 2695 HPLC system and Waters 2996 DAD detector (Waters Technologies, USA). The concentrations of glycyrrhizic acid, liquiritin and isoliquiritin were analyzed by chromatography on an octadecyl silica column (250 mm × 4.6 μm, Dikma, China) with isocratic elution of the mobile phase consisting of acetonitrile and deionized water with 0.1 % phosphoric acid at a flow rate of 1.0 mL/min. The column temperature was 30 °C and the detection wavelengths of glycyrrhizic acid, liquiritin and isoliquiritin were 250, 276 and 360 nm, respectively. A 10-μL quantity of the extract solution was injected into the HPLC system for analysis.

### UV methods

UV spectrophotometry was employed to analyze the content of licorice total flavonoids. The UV method was implemented on an Agilent 8450 UV spectrophotometer with a quartz cuvette (Agilent Technologies, USA). The analysis of licorice total flavonoids was as follows. A 0.5-mL quantity of 10 % KOH was used to prepare different diluted solutions. Reactions proceeded for 60 min in 5-mL volumetric flasks. The detection wavelength of licorice total flavonoids was 335 nm.

### Software and data analysis

Data analysis was performed by the Unscrambler 9.6 software package (CAMO Software AS, Norway), VISION software (Foss NIR System, Silver Spring, MD, USA) and MATLAB software (MATLAB v7.0, The Math Works, MA, USA). MWPLS and SiPLS algorithms used in this paper were downloaded from http://www.models.kvl.dk/. Ninety-three samples were divided to 62 calibration samples and 31 validation samples by the Kennard–Stone (KS) algorithm [16, 17]. Additionally, the PLS, MWPLS and SiPLS models were evaluated according to chemometrics indicators. All three methods were based on the root-mean-square error (RMSE):

$$ RMSE = \sqrt {\frac{{\sum {_{i = 1}^{I} \left( {c_{i} - \hat{c}_{i} } \right)^{2} } }}{I}} , $$

where $c_{i}$ is the reference values of the extraction of *Gancao* detected by HPLC and UV analysis, $\hat{c}_{i}$ denotes the estimated values for different samples, $I$ is the number of samples in each set [18, 19].

## Results and discussion

### Quantitative analysis of glycyrrhizic acid, liquiritin and isoliquiritin by HPLC

The reference values of three compounds were given in (Table S2 in Additional file 1). The calibration curves of glycyrrhizic acid, liquiritin and isoliquiritin exhibited good linearity (R^2^ = 0.9990, R^2^ = 0.9995, R^2^ = 0.9990) with the linear range extending from 0.407 to 4.070 μg, from 0.108 to 1.085 μg and from 0.016 to 0.168 μg, respectively. The response precision (intermediate precision and repeatability), stability and accuracy (recovery) met the requirements of analysis.

### Quantitative analysis of total flavonoids by the UV method

The linear regression of licorice total flavonoids gave y = 97.323x + 0.0413 (R^2^ = 0.9992), with the linear range being 1.59–9.54 μg. The precision (intermediate precision and repeatability), stability and accuracy (recovery studies) of the UV method satisfied the demands of analysis. The minimum, maximum and average concentrations of licorice total flavonoids were 0.044, 1.914 and 0.753 mg/mL, respectively.

### NIR spectral characteristics

**Fig. 2:**
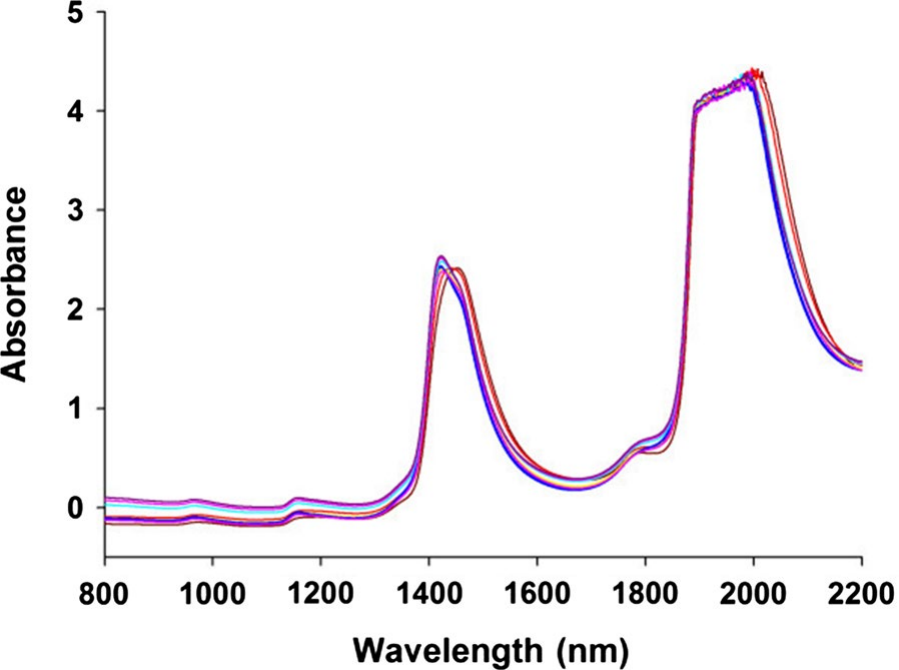
The NIR spectra of licorice

There was a large fluctuation in 2000–2200 nm because of a high level of noise in the combination region (Fig. 2). Additionally, aqueous solution is intensely absorbed at 1950 nm [20, 21]. There are large signal fluctuations in the spectral region of 780–2100 nm, suggesting that this spectral region contained the main information on concentrations. Furthermore, variable selection was selected by MWPLS and SiPLS method to obtain multivariable models.

### Optimum result of NIR pretreatment methods and latent factors

**Fig. 3:**
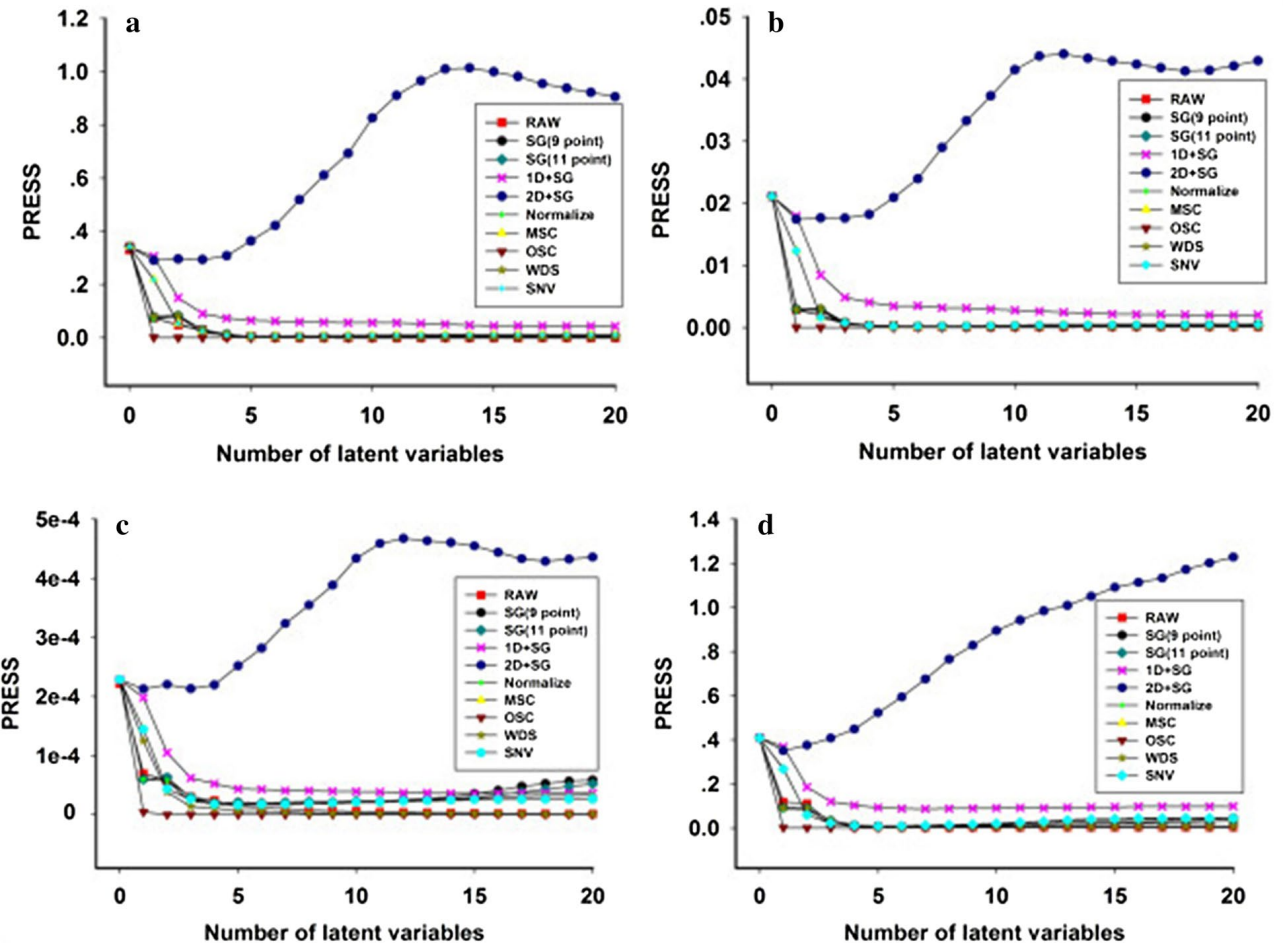
Predicted residual sum of squares (PRESS) plot of PLS model by different pretreatments

The spectra were affected by spectral noise, baseline drift and overlapping peaks. Spectral pretreatment methods were applied before the model was established to improve the accuracy of the model performance. Several pretreatment methods were applied to the spectral data set. The raw spectra, 11-point Savitzky–Golay and first derivative (SG + 1D) spectra, 11-point Savitzky–Golay and second derivative (SG + 2D) spectra, nine-point Savitzky–Golay (SG) spectra and 11-point SG spectra were thus compared in eliminating interference information [22]. The standard normal variation (SNV) and multiplicative scatter correction (MSC) were applied to reduce the effect of small particles in the extraction solution [23]. An orthogonal signal correction (OSC) was applied to pretreat the complex system [24]. Normalization was also applied before establishing the PLS model. Leave-one-out cross-validation was used to select an appropriate pretreatment method. The number of latent variable factors was investigated by leave-one-out cross-validation. The optimum number of latent factors was determined according to the lowest predicted residual sum of squares (PRESS) value [23]. Figure 3 shows the relationship between the latent variable and PRESS value for different pretreatment methods. OSC was found to be the best pretreatment method in terms of R^2^, RMSEC and RMSECV. Additionally, the nine-point SG, 11-point SG and raw spectra had low PRESS values. However, RMSEP and R_pre_^2^ of OSC were worse than those of other pretreatment methods (Table 1). Therefore, combining with the evaluation parameters, the raw spectra was selected to establish the PLS model for each quality parameter. According to the PLS results, the model performances achieved by MWPLS and SiPLS algorithms were compared to obtain low prediction error.

**Table 1:** PLS model performance with different pretreatments

Quality parameter	Pretreatments	Latent	Calibration set		Validation set		Prediction set	
		factors	RMSEC	R^2^	RMSECV	R^2^	RMSEP	R^2^
Glycyrrhizic	Raw	5	0.0364	0.9960	0.0453	0.9940	0.0479	0.9903
acid	SG (9)	5	0.0495	0.9926	0.0662	0.9872	0.1023	0.9556
	SG (11)	5	0.0496	0.9926	0.0657	0.9874	0.0447	0.9915
	SG (11) + 1D	5	0.0980	0.9710	0.2550	0.8100	0.2045	0.8225
	SG (11) + 2D	5	0.4151	0.4800	0.5397	0.1493	0.5121	0.1128
	MSC	4	0.0775	0.9819	0.0948	0.9738	0.0847	0.9695
	SNV	4	0.0783	0.9815	0.0955	0.9734	0.0861	0.9686
	OSC	1	0.0234	0.9984	0.0274	0.9978	0.1004	0.9587
	WDS	5	0.0484	0.9929	0.0620	0.9888	0.0379	0.9941
	Normalize	5	0.0381	0.9956	0.0607	0.9892	0.0495	0.9896
Liquiritin	Raw	4	0.0137	0.9909	0.0182	0.9844	0.0090	0.9958
	SG (9)	4	0.0138	0.9906	0.0163	0.9874	0.0121	0.9924
	SG (11)	5	0.0139	0.9906	0.0163	0.9874	0.0122	0.9923
	SG (11) + 1D	5	0.0225	0.9752	0.0587	0.8370	0.0465	0.8885
	SG (11) + 2D	5	0.1031	0.4798	0.1320	0.1746	0.1192	0.2671
	MSC	4	0.0146	0.9896	0.0180	0.9846	0.0158	0.9871
	SNV	4	0.0148	0.9893	0.0181	0.9845	0.0161	0.9866
	OSC	1	0.0050	0.9988	0.0058	0.9984	0.0161	0.9866
	WDS	4	0.0144	0.9899	0.0166	0.9869	0.0126	0.9918
	Normalize	3	0.0185	0.9832	0.0217	0.9778	0.0165	0.9859
Isoliquiritin	Raw	4	0.0038	0.9339	0.0044	0.9151	0.0029	0.9545
	SG (9)	4	0.0039	0.9302	0.0044	0.9134	0.0029	0.9554
	SG (11)	4	0.0004	0.9293	0.0045	0.9129	0.0029	0.9555
	SG (11) + 1D	5	0.0028	0.9652	0.0066	0.8081	0.0050	0.8636
	SG (11) + 2D	1	0.0111	0.4320	0.0146	0.0686	0.0130	0.0865
	MSC	4	0.0035	0.9441	0.0042	0.9224	0.0034	0.9388
	SNV	4	0.0035	0.9432	0.0042	0.9220	0.0034	0.9388
	OSC	1	0.0020	0.9988	0.0021	0.9987	0.0144	0.9169
	WDS	2	0.0058	0.9301	0.0062	0.9089	0.0070	0.9049
	Normalize	4	0.0038	0.9335	0.0044	0.9142	0.0032	0.9467
Total flavonoids	Raw	4	0.0717	0.9870	0.0785	0.9849	0.0484	0.9927
	SG (9)	5	0.0739	0.9862	0.0953	0.9778	0.0590	0.9891
	SG (11)	5	0.0734	0.9864	0.0956	0.9776	0.0922	0.9734
	SG (11) + 1D	5	0.1062	0.9752	0.2984	0.7823	0.1950	0.8811
	SG (11) + 2D	1	0.4615	0.4621	0.5937	0.1381	0.5538	0.0411
	MSC	4	0.0645	0.9895	0.0925	0.9791	0.0623	0.9879
	SNV	4	0.0902	0.9795	0.1084	0.9713	0.0973	0.9704
	OSC	1	0.0903	0.9794	0.1081	0.9715	0.0989	0.9695
	WDS	5	0.0412	0.9957	0.0450	0.9951	0.1480	0.9315
	Normalize	5	0.0722	0.9868	0.0890	0.9807	0.0539	0.9909

### Performance of the MWPLS model for the four compounds

The function of the MWPLS model can be briefly described as the selection of informative regions and the approximation of latent factors [13]. Different moving window sizes *H* were selected, and the RMSECV was calculated for the various window sizes and a various number of factors. If the MWPLS model was better than the PLS model, it would have a lower RMSECV than the PLS model. For the four compounds in licorice, the MWPLS model was established in the range from 800 to 2200 nm, a range corresponding to 2800 data. The size of the moving window *H* varied from 13 to 41.

**Fig. 4:**
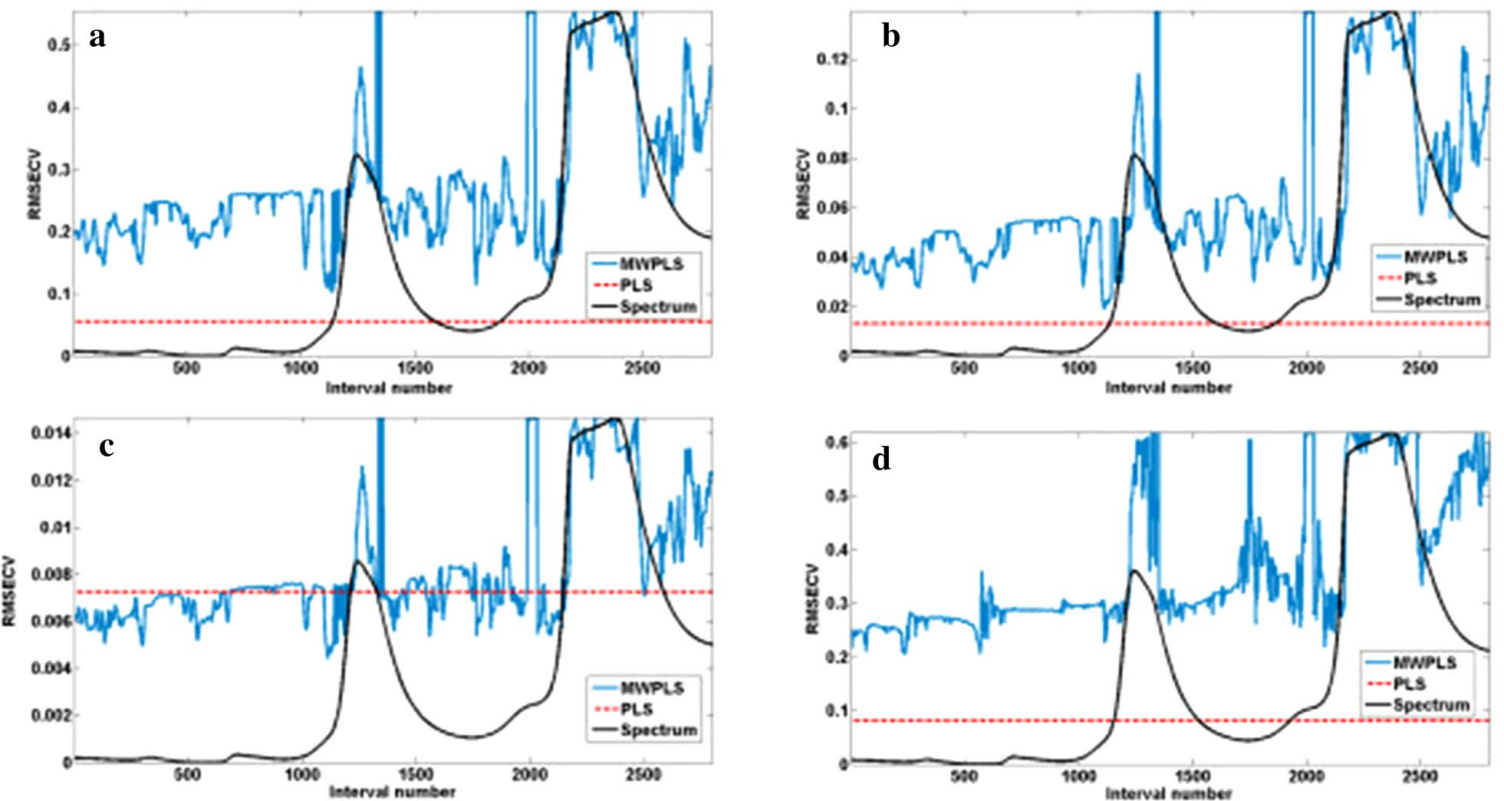
RMSECV for 35 windows size and 7 latent variables for the full-spectrum models: glycyrrhizic acid (**a**), liquiritin (**b**), isoliquiritin (**c**), total flavonoids (**d**)

Thus, moving windows were optimized with an RMSECV value lower than that for the PLS model [29]. The result demonstrated that RMSECV values for glycyrrhizic acid, liquiritin and licorice total flavonoids were all higher than those in the case of the full-spectrum PLS model, revealing that it was inappropriate to use MWPLS models for these three ingredients (Fig. 4). For isoliquiritin, the MWPLS model had the lowest RMSECV value, corresponding to *H* = 35. However, in contrast to the full-spectrum PLS model, the MWPLS model could not perform better for isoliquiritin, which might be attributed to the low content of isoliquiritin.

### Performance of the SiPLS model for the four compounds

The use of the SiPLS model was investigated as another variable selection method. The full spectrum was split into intervals. Several intervals constituted a joint model. The PLS was established for each joint model. The RMSECV value was regarded as a measurement of the accuracy of the model. The subinterval combination was selected on the basis of the combination of high accuracy of the joint model and a low RMSECV value. For the extraction of APIs, the optimal parameters of the SiPLS model were taken from the literature [25]. Each optimal SiPLS model was built by a combination of three subintervals taken from 20 equidistant subintervals.

**Fig. 5:**
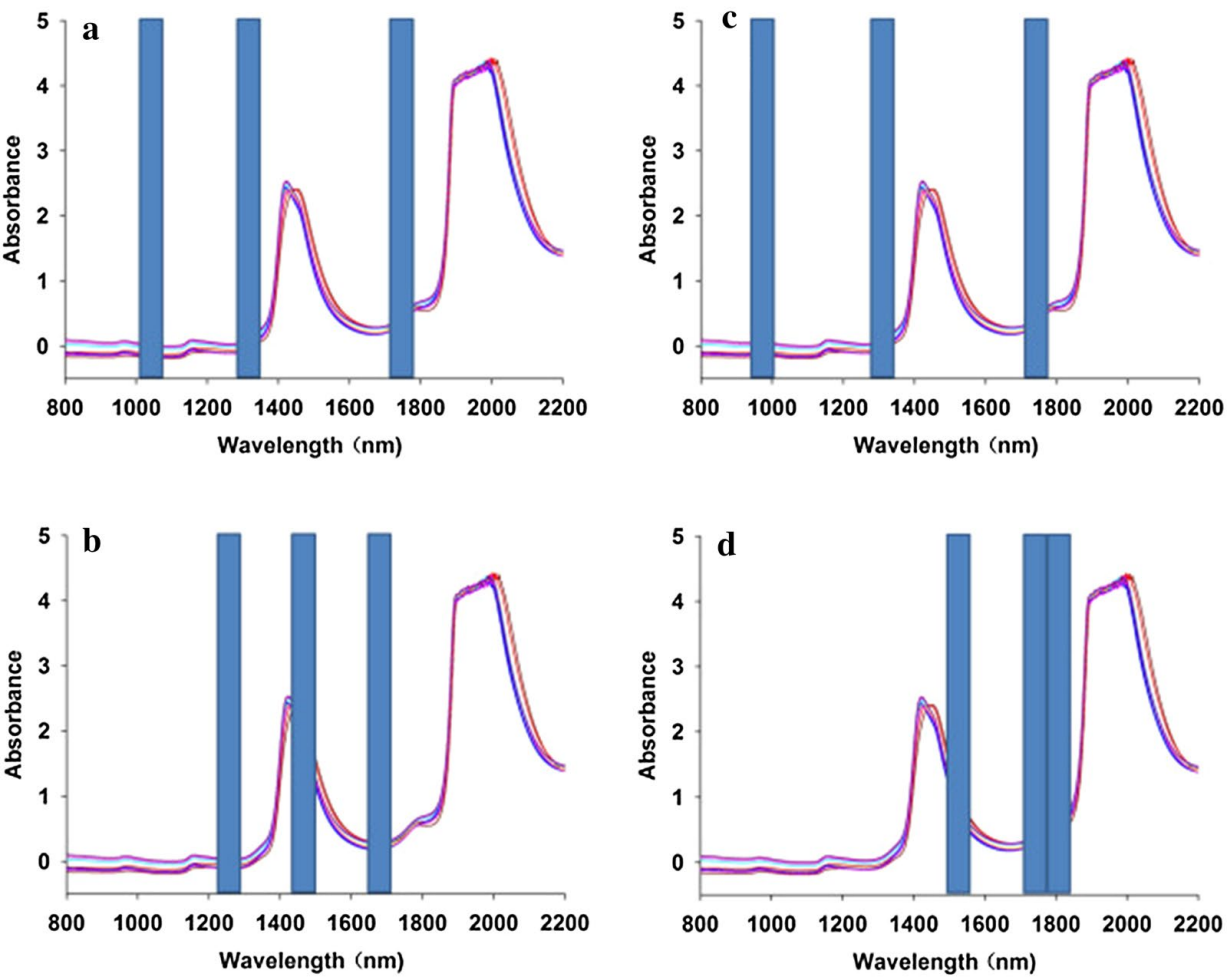
The optimum subinterval combination (*blue column*) selected by SiPLS for the quantitative determination of licorice acid (**a**), liquiritin (**b**), isoliquiritin (**c**), total flavonoids (**d**)

For glycyrrhizic acid, liquiritin, isoliquiritin and licorice total flavonoids, the optical subinterval combinations were respectively 1010–1080, 1290–1360, 1710–1780 nm; 940–1010, 1290–1360, 1710–1780 nm; 1220–1290, 1430–1500, 1640–1710 nm; and 1500–1570, 1710–1780, 1780–1850 nm, as shown by the three blue regions in Fig. 5. The RMSEC, RMSECV, and RMSEP values and corresponding R^2^ of the SiPLS model and PLS model are given in (Table 3 in Additional file 1). The performance results of the SiPLS and PLS models in calibration set were similar for the four compounds in licorice, but in the predicted sets of the compounds. The SiPLS model performed better than the PLS model. SiPLS models were thus established for the extraction of licorice.

### Performance of SiPLS models for the extraction of the four compounds

**Fig. 6:**
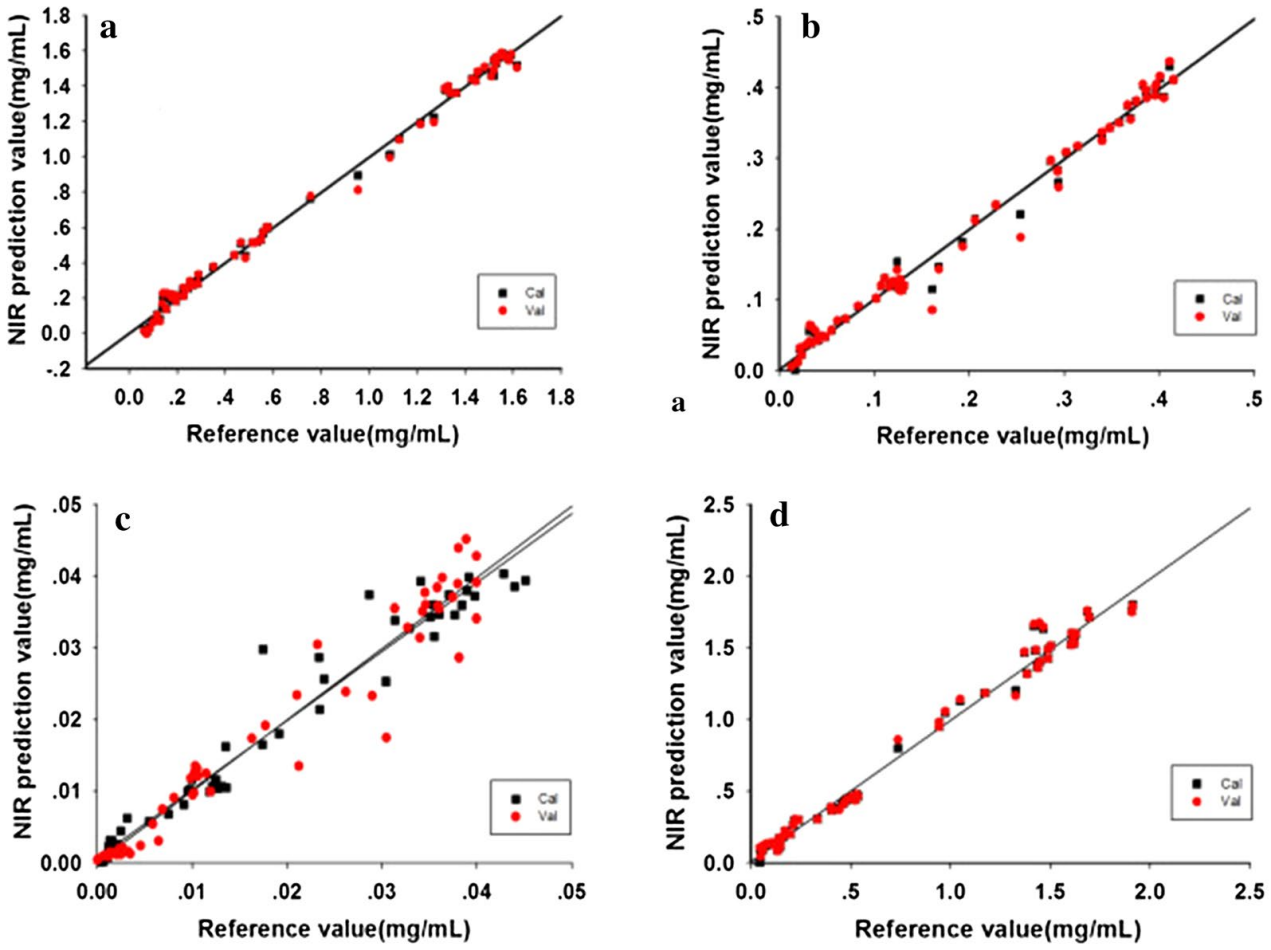
NIR predictions versus the reference method result, glycyrrhizic acid (**a**), liquiritin (**b**), isoliquiritin (**c**), licorice total flavonoids (**d**). Cal represents calibration set; Val represents validation set

The SiPLS method was used to establish models of extraction. R^2^ for glycyrrhizic, liquiritin and licorice total flavonoids mostly exceeded 0.98, indicating that the models had good accuracy. The RMSEC, RMSECV, and RMSEP were less than 0.07 for the four ingredients. Figure 6 presents the regression of calibration and the prediction result for each SiPLS model. The results showed that the reference value and predicted value almost aligned. However, for isoliquiritin, R^2^ was about 0.93, which can be attributed to the low content of isoliquiritin and high detection limit of NIR technology.

### SiPLS model assessment by relative errors and the F-values

The relative errors and F values were further employed to determine the predictive ability of the SiPLS model and to verify the reliability of the online NIR model in the extraction process for licorice. Different extraction phases of licorice for the four ingredients are shown in Table 2. As the contents of the four compounds (glycyrrhizic acid, liquiritin, isoliquiritin and total flavonoids) were different, and 93 samples were selected by the KS algorithm for each compound, the number of samples of each compound was different in the same phase. Although some samples could not be detected by HPLC and UV analyses, all results except those of the third extraction and isoliquiritin satisfied the needs of analysis. The mean relative error of the third extraction phase was higher than that of the first and second extraction phases. In the same extraction phase, the relative error of isoliquiritin was higher than that of other ingredients. These results could be attributed to the low concentration (micro analysis) of the third extraction and isoliquiritin.

**Table 2:** Relative errors of the contents of the four compounds in different extraction phases

Extraction	Sample ID	Relative error of compounds			
phases		Glycyrrhizic acid	Liquiritin	Isoliquiritin	Total flavonoids
1st extraction	E_1a_	1.82 %	1.72 %	8.84 %	0.86 %
	E_1b_	1.69 %	3.46 %	12.25 %	3.19 %
	E_1c_	2.28 %	0.97 %	1.78 %	3.17 %
	E_1d_	3.17 %	2.41 %	16.86 %	0.06 %
	E_1e_	5.65 %	3.38 %	0.58 %	1.48 %
	E_1f_	5.22 %	5.15 %	3.86 %	7.75 %
	E_1g_	6.01 %	4.76 %	15.47 %	3.75 %
	E_1h_	1.78 %	3.10 %	38.09 %	3.93 %
	E1i	5.54 %	4.99 %	NA	NA
	E_1j_	1.51 %	NA	NA	NA
	E_1k_	2.76 %	NA	NA	NA
	E_1average_	3.40 %	3.33 %	12.22 %	3.02 %
2nd extraction	E_2a_	2.78 %	3.09 %	4.76 %	2.88 %
	E_2b_	4.29 %	3.30 %	29.87 %	6.78 %
	E_2c_	0.04 %	10.11 %	36.90 %	9.42 %
	E_2d_	6.36 %	8.65 %	15.08 %	9.49 %
	E_2e_	0.81 %	5.88 %	5.46 %	15.34 %
	E_2f_	4.46 %	14.83 %	5.44 %	11.11 %
	E_2g_	0.85 %	1.55 %	36.67 %	13.23 %
	E_2h_	3.36 %	4.04 %	0.09 %	17.39 %
	E_2i_	0.83 %	1.24 %	2.97 %	14.29 %
	E_2j_	12.58 %	19.25 %	8.48 %	11.51 %
	E_2k_	5.31 %	5.16 %	4.78 %	10.28 %
	E_2l_	4.29 %	15.46 %	4.64 %	2.86 %
	E_2m_	13.78 %	5.71 %	22.78 %	3.04 %
	E_2n_	2.30 %	12.58 %	18.42 %	6.68 %
	E_2h_	NA	NA	1.61 %	9.11 %
	E_2average_	4.42 %	7.92 %	13.20 %	9.56 %
3rd extraction	E_3a_	51.19 %	14.08 %	4.01 %	9.60 %
	E_3b_	4.64 %	11.55 %	92.73 %	2.82 %
	E_3c_	7.92 %	22.88 %	78.88 %	2.76 %
	E_3d_	1.65 %	10.59 %	55.67 %	21.92 %
	E_3e_	18.76 %	7.47 %	74.38 %	12.41 %
	E_3f_	6.53 %	25.21 %	99.38 %	22.22 %
	E_3g_	NA	5.99 %	3.78 %	34.93 %
	E_3h_	NA	10.84 %	49.16 %	10.74 %
	E_3average_	13.59 %	12.95 %	52.35 %	14.11 %

*NA* Samples were not selected by the KS algorithm in this phase

In addition, the NIR and reference methods were compared using an F test [26]. The F values of glycyrrhizic acid, liquiritin, isoliquiritin and total flavonoids were 10,765, 32,431, 649 and 6080 respectively (*P* < 0.01). According to the F value distribution table, for a significance level $\partial = 0.01$ and number of samples n = 93, the F value is 6.90 (*P* < 0.01). The F values of the four compounds given above were much higher than 6.90 (*P* < 0. 01), showing the significant relationship between the prediction value and reference value. Furthermore, multivariate detection limit (MDL) values were proposed in evaluating the model according to the type of errors and concentration ranges [27]. The MDL was almost 14 ppm, confirming that the online NIR platform could detect low amounts of CM.

## Conclusion

The study demonstrated the feasibility of online NIR analysis in the multi-ingredient and multi-phase extraction of APIs from licorice.

## Additional file

10.1186/s13020-015-0069-2 **Table S1.** The sampling intervals in different extraction phases. **Table S2.** The HPLC results of different indicators. **Table S3.** The evaluation parameters of PLS and SiPLS models.

## Abbreviations

NIR: near infrared
API: active pharmaceutical ingredient
HPLC: high-performance liquid chromatography
UV: ultraviolet
KS: Kennard–Stone
OSC: orthogonal signal correction
WDS: wavelet denoised spectrum
PLS: partial least squares
MWPLS: moving-window partial least squares
SiPLS: synergy interval partial least squares
RMSEC: root-mean-square error of correction
RMSECV: root-mean-square error of cross-validation
RMSEP: root-mean-square error of prediction
TCM: traditional Chinese medicine
SG: Savitzky–Golay
1D: first derivative
2D: second derivative
SNV: standard normal variation
MSC: multiplicative scatter correction
PRESS: predicted residual sum of squares
