# Supplementary material for: Online near-infrared analysis coupled with MWPLS and SiPLS models for the multi-ingredient and multi-phase extraction of licorice (Gancao)
Source: Chin Med. 2015 Dec 18;10:38. doi: 10.1186/s13020-015-0069-2 (PMC4683800; doi:10.1186/s13020-015-0069-2)
Supplement: Supplementary file 1 — 10.1186/s13020-015-0069-2 Table S1. The sampling intervals in different extraction phases. Table S2. The HPLC results of different indicators. Table S3. The evaluation parameters of PLS and SiPLS models. [file 13020_2015_69_MOESM1_ESM.docx]

**Online near-infrared analysis coupled with MWPLS and SiPLS models for the multi-ingredient and multi-phase extraction of licorice (*Gancao)***

Yang Li^1,2,3^, Mingye Guo^1^, Xinyuan Shi^1,2,3^, Zhisheng Wu^1,2,3^*, Jianyu Li^1,2,3^, Qun Ma^1,4^, Yanjiang Qiao^1,2,3^*

^1^School of Chinese Materia Medica, Beijing University of Chinese Medicine, Beijing, China.

^2^Pharmaceutical Engineering and New Drug Development of Traditional Chinese Medicine, Ministry of Education, Beijing, China.

^3^Key Laboratory of Traditional Chinese Medicine-Information Engineering, State Administration of Traditional Chinese Medicine, Beijing, China.

^4^Beijing Key Laboratory for Basic and Development Research on Chinese Medicine, Beijing, China.

*Corresponding authors:

Zhisheng Wu and Yanjiang Qiao

Key Laboratory of TCM-information Engineering of State Administration of Traditional Chinese Medicine

Beijing

China

Email addresses:

YL: limingyangsoul@163.com

MYG: mingyeguo@163.com

XYS: shixinyuan01@163.com

ZSW: wzs@bucm.edu.cn

JYL: bucmljy@sina.com

QM: maqun99@126.com

YJQ: yjqiao@263.net

Table S1 The sampling intervals in different extraction phases

| Extraction phases | Extraction time | | | |
| --- | --- | --- | --- | --- |
|  | Heating | 0-1h | 1-2 h | 2-2.5 h |
| 1st extraction | 4 min | 4 min | 5 min | 10 min |
| 2nd extraction | 5 min | 5 min | 5 min | 10 min |
| 3rd extraction | 5 min | 10 min | 10 min | 10 min |

Table S2 The HPLC results of different indicators

| Quality parameters | Sample  number | Minimum value | Maximum value | Mean | Standard deviation |
| --- | --- | --- | --- | --- | --- |
| Glycyrrhizic acid (mg/mL) | 93 | 0.0631 | 1.6169 | 0.7610 | 0.553 |
| Liquiritin (mg/mL) | 93 | 0.0127 | 0.4155 | 0.1756 | 0.139 |
| Isoliquiritin (mg/mL) | 93 | 0.0004 | 0.0451 | 0.0165 | 0.015 |

Table S3 The evaluation parameters of PLS and SiPLS models

|  |  | Latent | Calibration set | | Validation set | | Prediction set | |
| --- | --- | --- | --- | --- | --- | --- | --- | --- |
| Component | Models | factors | RMSEC | R^2^ | RMSECV | R^2^ | RMSEP | R^2^ |
| Glycyrrhizicacid | PLS | 5 | 0.0364 | 0.9960 | 0.0453 | 0.9940 | 0.0479 | 0.9903 |
|  | SiPLS | 5 | 0.0367 | 0.9960 | 0.0456 | 0.9940 | 0.0089 | 0.9958 |
| Liquiritin | PLS | 4 | 0.0137 | 0.9909 | 0.0182 | 0.9844 | 0.0090 | 0.9958 |
|  | SiPLS | 4 | 0.0138 | 0.9908 | 0.0182 | 0.9919 | 0.0089 | 0.9986 |
| Isoliquiritin | PLS | 4 | 0.0038 | 0.9339 | 0.0044 | 0.9151 | 0.0029 | 0.9545 |
|  | SiPLS | 4 | 0.0034 | 0.9465 | 0.0039 | 0.9331 | 0.0033 | 0.9365 |
| Total flavonoids | PLS | 4 | 0.0717 | 0.9870 | 0.0785 | 0.9849 | 0.0484 | 0.9927 |
|  | SiPLS | 4 | 0.0723 | 0.9870 | 0.0792 | 0.9849 | 0.0454 | 0.9926 |
